# Exploring automatic inconsistency detection for literature-based gene ontology annotation

**DOI:** 10.1093/bioinformatics/btac230

**Published:** 2022-06-27

**Authors:** Jiyu Chen, Benjamin Goudey, Justin Zobel, Nicholas Geard, Karin Verspoor

**Affiliations:** School of Computing and Information Systems, The University of Melbourne, Parkville, VIC 3010, Australia; School of Computing and Information Systems, The University of Melbourne, Parkville, VIC 3010, Australia; School of Computing and Information Systems, The University of Melbourne, Parkville, VIC 3010, Australia; School of Computing and Information Systems, The University of Melbourne, Parkville, VIC 3010, Australia; School of Computing and Information Systems, The University of Melbourne, Parkville, VIC 3010, Australia; School of Computer Technologies, RMIT University, Melbourne, VIC 3000, Australia

## Abstract

**Motivation:**

Literature-based gene ontology annotations (GOA) are biological database records that use controlled vocabulary to uniformly represent gene function information that is described in the primary literature. Assurance of the quality of GOA is crucial for supporting biological research. However, a range of different kinds of inconsistencies in between literature as evidence and annotated GO terms can be identified; these have not been systematically studied at record level. The existing manual-curation approach to GOA consistency assurance is inefficient and is unable to keep pace with the rate of updates to gene function knowledge. Automatic tools are therefore needed to assist with GOA consistency assurance. This article presents an exploration of different GOA inconsistencies and an early feasibility study of automatic inconsistency detection.

**Results:**

We have created a reliable synthetic dataset to simulate four realistic types of GOA inconsistency in biological databases. Three automatic approaches are proposed. They provide reasonable performance on the task of distinguishing the four types of inconsistency and are directly applicable to detect inconsistencies in real-world GOA database records. Major challenges resulting from such inconsistencies in the context of several specific application settings are reported. This is the first study to introduce automatic approaches that are designed to address the challenges in current GOA quality assurance workflows. The data underlying this article are available in Github at https://github.com/jiyuc/AutoGOAConsistency.

## 1 Introduction

Literature-based gene ontology annotation (GOA) (Carbon *et al.*, 2021) is the representation of gene product functions in terms of controlled vocabulary descriptors, based on information reported in the primary literature (Ashburner *et al.*, 2000). GOA provides a snapshot of current knowledge about specific gene products. Such annotations support a wide range of computational biomedical research tasks, including gene-category analysis (Bauer, 2017) and gene enrichment analysis (Huang *et al.*, 2009). More recent annotations within databases have been found to provide more informative results than earlier annotations during gene enrichment analysis (Kramarz *et al.*, 2020). This may be because more recent annotations and published literature have higher consistency and are more current and specific in representing gene function knowledge.

Several quality issues have been reported to occur in biological databases, including redundancy, inconsistency, or errors (Bateman *et al.*, 2021; Bult *et al.*, 2019; Carbon *et al.*, 2021; Chen *et al.*, 2017a,b). Faria *et al.* (2012) estimated that 23% of the proteins and 83% of protein functions in the UniProtKB database (Bateman *et al.*, 2021) have inconsistent GOA when considering orthologous or homologous genes. Both electronically inferred and literature-based GOA have been found to utilize broad GO terms without sufficient representation of specificity in gene functional information (Camon *et al.*, 2005; Škunca *et al.*, 2012), violating the GOA principle that GO terms should be grounded in the most granular level supported by evidence (Poux and Gaudet, 2017). In prior work, Chen *et al.* (2021) formalized the typology of three types of term-inconsistency and one type of code-inconsistency in literature-based GOA. They proposed a text mining model for discriminating consistency and different kinds of inconsistency on a synthetic dataset. However, their implementation requires the manual pre-extraction of evidence sentences from full-text articles. Thus, it is not directly applicable to GOA records in real-world databases that point only to a PubMed identifier.

Consistency review of GOA has been prioritized in quality maintenance routines by the GO curation community (Carbon *et al.*, 2021). However, existing approaches are neither efficient nor directly applicable to assuring literature-based GOA consistency. A purely manual approach, relying on comprehensive guidelines and time-consuming quality review of primary literature by professional curators (Balakrishnan *et al.*, 2013; Poux and Gaudet, 2017; Thomas, 2017), is costly and does not scale with rapidly evolving gene function knowledge. Automatic approaches are needed to improve the efficiency of GOA maintenance. However, existing automatic approaches are not directly applicable to the detection of inconsistency in existing records that are currently stored within databases, but rather focus on the task of adding new GOA records into databases. For example, the document triage system TextPresso (Müller *et al.*, 2018) improves the efficiency of GOA production by automatically retrieving only a subset of publications relevant to the annotation of a certain gene product for human curators.

An automated dictionary-based concept recognition tool such as ConceptMapper (Tanenblatt *et al.*, 2010) can improve the efficiency of GOA production. This tool has achieved competitive performance in annotating GO concepts over the Colorado Richly Annotated Full Text (CRAFT) corpus (Bada *et al.*, 2012; Funk *et al.*, 2014). However, it cannot recognize GO concepts that do not explicitly occur as phrases within evidence texts. For example, ‘positive regulation of vesicle fusion (GO: 0031340)’ cannot be recognized from ‘Rat SYT1 gave rise to efficient Ca2+-promoted fusion activity’.

To date, no tools have been implemented to evaluate the consistency between gene mentions in experimental statements and the gene product annotated in GOA. For example, given a GOA with an experimental statement that contains two gene mentions ‘RHGF-2 RhoGEF activity is specific to the *Caenorhabditis elegans* RhoA homolog RHO-1 as determined by direct binding, GDP/GTP exchange and serum response element-driven reporter activity. (PMID: 22363657)’ and the GO term ‘GDS (GO: 0005085)’, a consistent GOA should associate the term with gene ‘RHGF-2 (GeneID: 173748)’, not gene ‘rho-1 (GeneID: 178458)’.

GOA is an important resource for supporting modern biological research. However, GOA is subject to inconsistencies such as the selection of overly shallow GO terms or incorrect association to gene mentions. These inconsistencies are problematic because they can influence the reliability of downstream biological research tasks or cause cascading errors in biological databases (Gilks *et al.*, 2002). There is no existing approach to automatically detect GOA inconsistency. To address this issue, we have undertaken an initial feasibility study to explore and characterize four major types of inconsistencies in literature-based GOA and propose automatic methods for the detection of inconsistency grounded at record level. We focus on a particular type of GOA in which the evidence is experimental statements within primary literature and is tagged with experimental evidence codes (http://geneontology.org/docs/guide-go-evidence-codes/) (EXP, IDA, IPI, IMP, IGI, IEP).

**Fig. 1.**
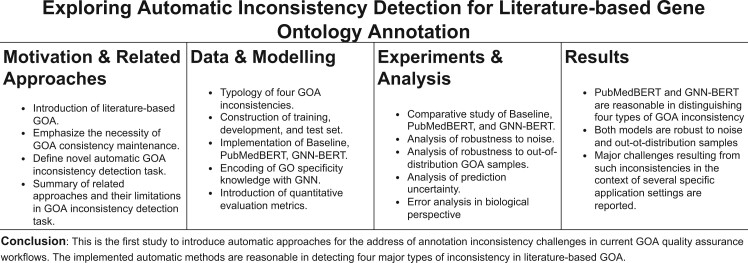
Visual abstract

To the best of our knowledge, this is the first study that empirically explores the feasibility of automatic methods for real-world literature-based GOA consistency assurance. We make three key contributions:

- We provide a definition for a novel GOA inconsistency detection task applicable for GO curation.
- We construct a dataset that simulates both self-consistent and four realistic types of inconsistent GOA instances derived from curation guidelines (Balakrishnan *et al.*, 2013; Carbon *et al.*, 2021; Poux and Gaudet, 2017; Thomas, 2017), extending the heuristic rules proposed by Chen *et al.* (2021). Each inconsistent instance is synthesized using real-world GOA records sampled from either the NCBI gene database (Brown *et al.*, 2015) or the curator-validated BC4GO corpus (Van Auken *et al.*, 2014).
- Several automatic approaches are proposed. Evaluation over our dataset shows that they achieve reasonable performance in classifying inconsistencies in GOA, thus demonstrating that automatic methods are feasible and of value. An overview of this article is visually demonstrated in Figure 1.

## 2 Approach

We model GOA self-consistency or inconsistencies at record level as typed semantic relationships in a triplet of GO term, gene product and naturally written evidence, extending prior work where the gene product was not explicitly included (Chen *et al.*, 2021). Our approach is directly applicable for the detection of inconsistencies in GOA, also extending the previous approach that required (manually) pre-extracted short sentence as input. We aim to develop classification models that, for a given GOA triplet as input, make a classification decision as to whether the triplet is self-consistent, and if not, what type of inconsistency is reflected in the instance. That is, the classifier makes a determination about whether the textual evidence supports the GO term annotation to the given gene product. To simplify our study, we initially focus on exploring feasible implementations that aim to detect the primary single type of inconsistency in each record. Hence, we assume that a GOA can only be in one type of inconsistency, and further that the typed inconsistencies are independent of each other.

We first review methods applicable to infer the semantic relationship between naturally written text pieces, or to quantify ontological biological knowledge as vectors. We then introduce our proposed automatic GOA inconsistency detector extended from these approaches.

### 2.1 Language models

Distributional language models such as PubMedBERT (Gu *et al.*, 2020) are potentially applicable to GOA consistency assessment. PubMedBERT has achieved competitive performance in semantic relation inference between short pieces of text in the biological domain, such as for the extraction of drug–drug interactions (Herrero-Zazo *et al.*, 2013), gene–disease associations (Becker *et al.*, 2004) and sentence-pair similarity estimation (Soğanc ioğlu *et al.*, 2017). However, these models have difficulties in discriminating between semantic relatedness and similarity (Kolb, 2009), limiting their ability to capture the nuanced semantics of related GO terms such as ‘feeding behavior (GO: 0007631)’ and ‘drinking behaviors (GO: 0042756)’. Besides, the input of PubMedBERT must be either a single or a pair of short text pieces while GOA involves a triplet of biological entities. The relatively long length of title and abstract texts may also negatively impact PubMedBERT’s reliability for semantic inference (cf. short sentence with 20 words or less).

### 2.2 GO semantic structure

Inference of GOA consistency requires a reference to the GO-directed acyclic graph (DAG) to make use of knowledge of GO specificity. This DAG can be modeled as a non-Euclidean heterogeneous graph where GO terms are vertices and hierarchical relations are typed edges. For example, the vertex ‘programmed cell death (GO: 0012501)’ is connected to the vertex ‘cell death (GO: 0008219)’ by ‘is_a’ typed edge. A single GO term does not have a clear specificity level, given that there may be multiple paths from the term to the root. Specificity is also only computable for a pair of directly or indirectly connected GO terms. For example, we cannot infer the specificity of gene function knowledge between two children of ‘feeding behavior (GO: 0007631)’, ‘salt aversion (GO: 0035199) and ‘drinking behavior (GO: 0042756)’, as they are disconnected on the GO DAG. Hamilton *et al.* (2017) developed GraphSAGE by extending the graph neural network (GNN) model to represent the semantics of vertices through the iterative aggregation of features in neighboring vertices. GraphSAGE is based on inductive learning and thus can learn a set of universal parameters across non-overlapping sub-graphs to encode structural information. These characteristics are suitable for quantifying the GO specificity relations as new terms are continuously added into the vocabulary. However, the suitability of this architecture for encoding GO specificity knowledge needs to be explored.

### 2.3 Transfer learning

An effective method for GOA consistency assurance requires the inference of both GO distributional semantics and knowledge of specificity. Transfer learning, a machine learning method where a model developed for a given task is reused as the starting point for a second task (Pan and Yang, 2010), is potentially suitable. Among other examples, Hu *et al.* (2019) proposed the use of transferred node representations learned from a biomedical knowledge graph to infer protein–protein interactions. Fout *et al.* (2017) used transferred structural information about proteins to predict protein interfaces. Zitnik *et al.* (2018) transferred the interactions between drugs and disease to another task in predicting polypharmacy side effects. Thus, it might be feasible to transfer quantified GO hierarchical knowledge to the task of GOA consistency assurance.

However, these approaches can easily encounter negative transfer (Rosenstein *et al.*, 2005), in which features transferred from a different task can lead to significant drops in performance. The presence of out-of-distribution samples is another challenge in transfer learning (Hu *et al.*, 2019). The semantics of a GO term can be different between different species, such as human and rats, which can lead to a non-uniform representation of the same GO term. It is infeasible to train a GOA consistency assurance model on all known existing species while new species are being annotated. Thus, the applicability of transferring a model from one learned species to new species is unclear.

### 2.4 GOA inconsistency modeling

We set up a rule Baseline using ConceptMapper (Tanenblatt *et al.*, 2010) and implement two machine-learning-based approaches to support GOA inconsistency classification through analysis of the evidence texts. One model is derived from fine-tuning the PubMedBERT distributional language model (Gu *et al.*, 2020) on the GOA inconsistency dataset. A novel model, GNN-BERT, is implemented through transfer learning and the combination of GO specificity knowledge and PubMedBERT.

We find that the rule Baseline can discriminate between over-specific and over-broad GOA inconsistencies. Two machine-learning-based approaches provide reasonable performance in discriminating all four types of inconsistency and are robust to noisy samples in the training set. The implementation using transfer learning does not appear to suffer from the negative transfer, while the implementation without transfer learning has higher confidence over most types of predictions on the test set. However, the machine-learning-based approaches are challenged by out-of-distribution samples due to different species across the training and test sets. They may also fail on unusually shallow or deep GO terms, or when related genes are provided as input.

## 3 Methods

### 3.1 Data

The NCBI provides a gene2go dataset through the GO Annotation File (http://geneontology.org/docs/download-go-annotations/), updated daily. We randomly sampled 5000 GOA records from gene2go where the associated evidence is a PubMed identifier (PMID) and the evidence code is experiment type. These GOA records are considered as the most reliable while the true scale of inconsistency (noise) is unknown. To simplify the study, we initially assume that all sampled GOA records are consistent and manipulate these via a targeted noise injection strategy to empirically analyze the impact of noise to the automatic implementations. We formalize four major types of inconsistencies including over-specific (OS), over-broad (OB), irrelevant GO mention (IM), incorrect association of gene product (IG) through the inference of database curation guidelines, quality reports and informal discussion with database curators. We synthesize each type of inconsistency by extending the heuristic rules proposed by Chen *et al.* (2021). Examples of this synthesis process are shown in Table 1 for each type of inconsistency. We randomly sample 20 000 instances to form a training set and 250 instances to form a development set. Each dataset is balanced with an equal number of self-consistent instances and the relevant type of inconsistency.

**Table 1.** Example of consistent GOA in BC4GO corpus annotated by expert curators and four major types of inconsistent GOA; in each case we show the synthetic strategy for modifying a consistent instance to generate an inconsistency

**Consistent GOA (CO)**
*Evidence:* Only a subset of *syt* isoforms stimulated SNARE-catalyzed fusion in the presence of Ca2+: *syt I–III, V–VII and IX–X*. (Remaining texts of title and abstract identified by (PMID: 18508778) are omitted.)
*GO term: response to calcium ion (GO: 0051592)*
*Gene: Syt1 (GeneID: 25716)*
**Over-specific GO term selection (OS)**
*GO term: cellular response to calcium ion (GO: 0071277)*
*Inconsistency:* The provided evidence does not indicate the stimulus of calcium ion lead to the change of state or activity of cell. Thus, the annotated GO term is over-specific.
*Synthesis:* Retrieve and randomly select the direct children with either ‘is_a’ or ‘part_of’ relation to replace the GO term in the consistent GOA instance on GO DAG. If no children are retrieved for a given consistent GOA, the synthesize of its over-specific inconsistency will be skipped.
**Over-broad GO term selection (OB)**
*GO term: response to metal ion (GO: 00106038)*
*Inconsistency:* The evidence has specified the experimented metal ion is calcium ion. Thus, the annotated GO term is over-broad.
*Synthesis:* Retrieve and randomly select ancestors with either ‘is_a’ or ‘part_of’ relation to replace the GO term in a consistent GOA instance on GO DAG. If no ancestor are retrieved for a given consistent GOA, synthesis of its over-broad inconsistency will be skipped.
**Irrelevant GO Mention (IM)**
*Evidence:* At anaphase, *Mtor(GeneID: 36264)* plays a role in *spindle* elongation, thereby affecting normal chromosome movement. (PMID: 19273613)
*GO term: spindle (GO: 0005819)*
*Inconsistency:* Although the GO term ‘spindle’ is mentioned as a keyword in the evidence text, it does not indicate gene ‘Mtor’ is localized at cell spindle. Thus, the annotation is an irrelevant GO mention.
*Synthesis:* Apply ConceptMapper (Funk *et al.*, 2014) to recognize GO mention from the text in title and abstract identified by the associated PMID in each consistent GOA instance. Then, randomly select a GO mention that was not annotated to the gene product in that PMID. Use the selected GO mention to replace with the original GO term to synthesize irrelevant GO mention inconsistency.
**Incorrect association of gene product (IG)**
*Evidence: RHGF-2* RhoGEF activity is specific to the *C.elegans* RhoA homolog *RHO-1* as determined by direct binding, GDP/GTP exchange and serum response element-driven reporter activity. (PMID: 22363657)
*GO term: GDS (GO: 0005085)*
*Gene: rho-1 (GeneID: 178458)*
*Inconsistency:* The evidence indicates guanyl-nucleotide exchange factor activity was associated with gene ‘RHGF-2(GeneID: 173748)’ instead of gene ‘rho-1(GeneID: 178458)’. Thus, the annotation is an incorrect association of gene product.
*Synthesis:* Retrieve all gene products and GO terms that associated with the same PMID in the consistent GOA dataset. Iterate over every GO term and shortlist gene products that were not associated to the GO term. Randomly select a shortlisted gene product to pair with the GO term and PMID to form incorrect association of gene product inconsistency.

We use the BC4GO corpus to synthesize a silver-standard test set with little or no noise. The BC4GO corpus was created by eight expert curators from five different model organism databases for the GO annotation task in BioCreative IV (Van Auken *et al.*, 2014). In contrast to a mention-based GO corpus such as CRAFT (Bada *et al.*, 2012), BC4GO provides each GO annotation with traceable evidence grounded at the sentence level within a literature source. BC4GO mirrors the real-world GO curation scenario and contains GOA in real-world database format. Thus, we assume there is no noise within BC4GO regarding annotation inconsistency. We randomly sample instances from BC4GO where the associated evidence texts are within either the title or abstract of the associated article. The same inconsistency synthesis strategy (Table 1) is applied to generate inconsistent instances. Only a small fraction of instances are inconsistent in real-world databases, and, while the exact fraction is unknown, we wish to explore the impact of the existence of imbalance. We therefore create an imbalanced test set by re-sampling instances from the synthesized dataset. We sample 238 self-consistent instances and 48 instances of each type of inconsistency to form an imbalanced test set, 430 instances in total.

The three datasets provide a realistic GOA format where each instance is represented as a triplet of gene product (identified by NCBI GeneID, represented by gene symbol), GO term (identified by GOID) and evidence (identified by PMIDs). The three synthesized datasets are independent of each other as the associated PMIDs do not overlap. We only work with the text in title and abstract as evidence because many publications do not grant accessibility to full-text articles.

We also explore different models in terms of their ability to discriminate the subtle semantics of GO specificity. Thus, we synthesize over-specific instances with GO terms as directly connected children and over-broad instances with GO terms as an ancestor. An ancestor may not directly connect to a descendant on GO DAG thus can have greater semantic distance. As a result, over-specific instances are more challenging than over-broad instances to automatic semantic inference models.

### 3.2 Modeling

We set up a Baseline using ConceptMapper (Funk *et al.*, 2014) and heuristic rules. We implemented two machine-learning models extending PubMedBERT (Gu *et al.*, 2020) for our proposed GNN-BERT models. We empirically explore the feasibility of the implementations in discriminating the four major types of inconsistent GOA on the test set.

#### Baseline

We set up a two-step rule-based Baseline to (a) identify inconsistent GOA and (b) classify inconsistent GOA into certain types. The Baseline is implemented by using ConceptMapper to automatically extract a list of GO terms in the first step. These GO terms were explicitly mentioned as phrases in the evidence text of each instance. An instance is considered to be inconsistent if its associated GO term does not appear in the output of ConceptMapper. After identifying an inconsistent instance, two heuristic rules are set up in the second step to further classify this instance as either over-specific or over-broad:

- *Over-specific*. An instance is over-specific when its associated GO term is a descendant of **every** GO term with either ‘is_a’ or ‘part_of’ relation in the output of ConceptMapper.
- *Over-broad*. An instance is over-broad when its associated GO term is an ancestor of **one of** the GO terms with either ‘is_a’ or ‘part_of’ relation in the output of ConceptMapper.

This Baseline is not applicable to the detection of the ‘irrelevant GO mention’ inconsistency; all IM instances will be classified as consistent since their associated GO terms will appear in the output of ConceptMapper. This will result in no TP predictions for the detection of IM inconsistency. Also, this Baseline cannot identify the gene product inconsistency (IG) because ConceptMapper, as used here, does not recognize gene mentions from free text. An alternative option is to use PubTator (Wei *et al.*, 2013) to recognize gene mentions, which we will explore in future work.

#### Fine-tuning on PubMedBERT

We select PubMedBERT, pre-trained using the title and abstract of many PubMed articles (Gu *et al.*, 2020). We fine-tune PubMedBERT using a sentence pair classification head on the training set, with the objective of classifying an input sequence as self-consistent or (one type of) inconsistency. We form every GOA triplet as a structured input sequence in the following format: ‘[CLS] title || abstract || gene symbol [SEP] GO term [SEP]’. (‘||’ denotes flat joint of texts, ‘[CLS]’ and ‘[SEP]’ are the mandatory markers in BERT-based models for indicating paired input entities). Each input sequence can be seen as consisting of gene conditioned evidence and GO term. We apply the PubMedBERT AutoTokenizer to the input sequence to split it into tokens. Out-of-vocabulary words are tokenized into separate text pieces; for example, ‘polyc’ and ‘##omb’ are counted as two individual tokens. The average length of the tokenized input sequences is 334 tokens in the training set and a majority of them are under 350 tokens. Thus, we pad input sequences using ‘[PAD]’ or truncate them into a fixed length of 350 tokens. The outputs of PubMedBERT are vectors of logits in five-dimension. Finally, the arg max function is applied to each logit vector to decide the most likely (in)consistent class an input sequence belongs to.

#### Encoding of GO specificity knowledge

We model the GO as a DAG using the DGL toolkit (Wang *et al.*, 2019). A GO term is selected if it appears in either the training, development, or test set. Ancestors and direct children are retrieved using the QuickGO API (Binns *et al.*, 2009) if they have either ‘is_a’ or ‘part_of’ relations to the selected GO term. All retrieved GO terms are modeled as vertices on the graph. The relationships between each ancestor–child pair are modeled as directed edges typed with ‘is_a’ or ‘part_of’ label. Two additional directed edges are created as ‘parent_is_a’ and ‘parent_part_of’ to reverse all ‘is_a’ and ‘part_of’ edges. This will allow bidirectional aggregation of node features during node representation learning between ancestors and children. This graph includes 40 400 vertices and 1 045 432 edges in total. The features of each vertex are encoded into 120D vectors using PubMedBERT’s AutoEncoder.

**Fig. 2.**
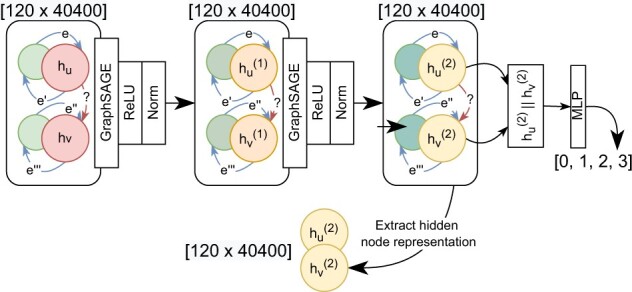
Architecture of graph neural network (GNN) with the objective of edge type classification for encoding GO specificity knowledge. $h_{u}^{n}$, the vector of vertex *u* in the *n*th layer of GNN; *e*, denotes the edge type vector; ||, flat concatenation of vectors; ReLU, rectified linear unit, the activation function; Norm, batch normalization; MLP, single-layered multi-layer perceptron

The encoding of GO specificity knowledge is learned from the objective of edge type classification on the constructed graph. We randomly partition and mask the labels of 770 979 edges from the graph as a training set and the remaining 274 453 edges as a test set. We use a GNN model (Fig. 2) to classify edge labels on the constructed graph, consisting of two GraphSAGE layers with pooling aggregation function in each layer (Hamilton *et al.*, 2017) and a single-layered multi-layer perceptron (MLP). The input and output of the two GraphSAGE layers are both 120 in dimension. The input of MLP is a 240-dimension vector, which is the flat concatenation of the two vertex vectors. Batch normalization is added after the activation functions in two GraphSAGE layers. We do not explore the alternative of adding normalization before the activation function (Ioffe and Szegedy, 2015) as this is out of the scope for our research. The output of the MLP layer is logit vectors in four dimensions. Finally, the arg max function is applied to each logit vector for deciding the most likely edge label that two connected vertices belong to.

We retrieve the specificity encoding of GO by extracting the vectors of vertices from the output of the last hidden layer ($h_{u}^{\left(2\right)},\;h_{v}^{\left(2\right)}$) after training with edge type classification. Table 2 shows the performance of GNN in classifying labels of test edges.

**Table 2.** Performance of GO-GNN on edge type classification

*Edge type*	*Precision*	*Recall*	*F*_1_
is_a	0.98	0.96	0.97
part_of	0.97	0.95	0.96
parent_is_a	0.97	0.96	0.96
parent_part_of	0.97	0.94	0.95

#### GNN-BERT for GOA inconsistency detection

**Fig. 3.**
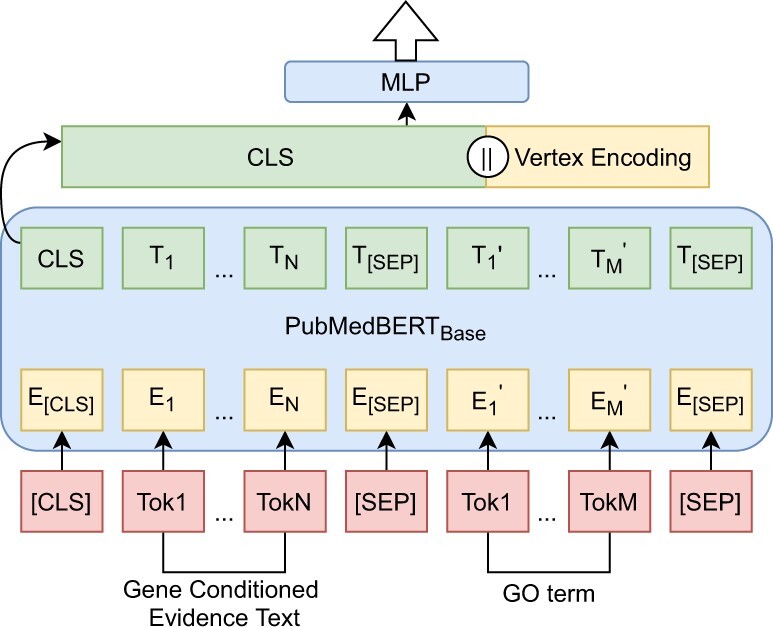
Architecture of GNN-BERT model for GOA (in)consistency detection. Tok* denotes a linguistic token, E* and T* denote a token embedding, *[CLS]* and *[SEP]* are special tokens marking the boundary of an input pair

We implement a GNN-BERT model (Fig. 3) that combines the distributional semantics and specificity knowledge of GO in each GOA instance for automatic GOA inconsistency detection. The combination is achieved by the flat concatenation of the specificity encoding of associated GO with the encoding of ‘[CLS]’ token in the last hidden layer of PubMedBERT for each instance. The concatenated vector will then be forwarded into a single-layered MLP, making the GNN-BERT as a single type multi-class classifier. The learning objective, input sequence format and operation to output logit vectors of GNN-BERT are exactly same as PubMedBERT. We assume GNN-BERT can outperform PubMedBERT in detecting GOA inconsistencies regarding GO specificity.

### 3.3 Metrics

We use *Precision* ($P=\frac{TP}{TP+FP}$), *Recall* ($R=\frac{TP}{TP+FN}$) and *F*_1_ score ($F_{1}=2\times \frac{P\times R}{P+R}$) as evaluation metrics. Evaluation follows the one-vs-all strategy for the detection of each specific type of inconsistency, e.g. assume positives are instances with over-specific label and negatives are instances with any other label. In this case, true positives (TP), false positives (FP) and false negatives (FN) are counted as follows:

- TP: The predicted label is over-specific for a positive instance
- FP: The predicted label is over-specific for a negative instance
- FN: The predicted label is not over-specific for a positive instance

Considering the number of instances is balanced across each type of inconsistency in the test set (48 instance in each type), we quantify a model’s overall performance by taking the average of the sum of *Precision*, *Recall* or *F*_1_ over each type of inconsistency (macro average). We focus on the evaluation of model’s discriminative power on different types of inconsistency. Thus, an instance will be treated as negative during evaluation if it is labeled as *consistent*. By doing so, we ensure that the evaluation is not biased by the number of consistent instances, as these are the majority class in real-world databases and the test set.

We quantify the uncertainty of model prediction using Shannon’s entropy; Equation 1, where *p_i_* denotes the probability that a GOA instance is of a given (in)consistency type *i*. Higher *τ* indicates greater uncertainty or lower confidence towards the predicted inconsistency label of an instance.

(1) $$\tau =-\sum_{i=0}^{4}p_{i}{\;\text{log}\;}_{2}p_{i},\tau \in (0,2.32).$$

### 3.4 Experiments

The best fine-tuned hyperparameter settings are summarized in Table 3 for PubMedBERT and GNN-BERT, using tricks suggested by Popel and Bojar (2018). PubMedBERT has two more fine-tuning epochs than GNN-BERT, giving current training set. We assume GNN-BERT converges faster than PubMedBERT due to its addition of informative GO specificity knowledge. The fine-tuning batch, warmup steps and weight decay are the same between two models. The values of these settings may slightly change when different training set are provided in future use. We design three experiments to explore the feasibility of two models.

**Table 3.** Hyperparameter settings for PubMedBERT and GNN-BERT during fine-tuning on the synthetic training set

*params*	PubMedBERT	GNN-BERT
Fine-tune epochs	5	3
Fine-tune batch	16	16
Warmup steps	300	300
Weight decay	0.01	0.01

#### Initial experiment

We directly apply the rule Baseline on the test set for classifying over-specific and over-broad inconsistencies as it does not need training. We fine-tune PubMedBERT and GNN-BERT on the synthesized training and development set and evaluate their performance on the test set.

#### Experiment on robustness to noise in the training set

We define noise of GOA (in)consistency as instances that are incorrectly labeled as consistent type but are actually a type of inconsistency, or vice versa. To simplify our study, we regard mis-annotations between different types of inconsistency as noise and focus on model robustness in discriminating all types of inconsistency as a whole from consistency. Precisely quantifying the amount of noise in either NCBI-gene2go resources or our synthesized dataset is infeasible as doing so would require manual curation. Thus, we propose an alternative strategy, designing a noise injection strategy to analyze the model’s robustness in detecting inconsistencies in the test set based on controlled manipulation of noise during the fine-tuning stage. We generate 8 noise-injected training sets by randomly relabeling 0, 10, 15, 25, 40, 60, 80 and 100% of instances in the original training set. The relabeling strategy involves the alternation of either one type of inconsistency label into consistent, or vice versa. Each instance is relabeled only once. Eight groups of PubMedBERT and GNN-BERT are independently fine-tuned on the right noise-injected datasets and evaluated on the same test set.

#### Experiment on robustness to out-of-distribution data

We define a GOA instance in the training set as an out-of-distribution (OOD) sample if the species of the associated gene product is excluded in the test set. A GOA instance in the training set as an in-distribution (ID) sample if the species of the associated gene product is shared with instances in the test set. We analyze the impact of fine-tuning with OOD samples on the performance of PubMedBERT and GNN-BERT. The test set consists of four species including fruit fly (taxonid: 7227), rat (taxonid: 10116), *Arabidopsis thaliana* (taxonid: 3702) and *C.elegans* (taxonid: 6239). All instances in the test set are in-distribution samples to the training set. We synthesize a new training set that contains only samples OOD w.r.t. the test set, consisting of 20 000 instances where all instances are associated with gene products in human (taxonid: 9606). Two groups of PubMedBERT and GNN-BERT models are independently fine-tuned on the original training set and OOD training set, and evaluated on the same test set. The rule Baseline is excluded in this experiment as it does not require fine-tuning during application thus will naturally not be influenced by OOD samples in the training set.

#### Correlation between prediction uncertainty and performance

We consider automatic models to be feasible in real-world application settings if they can quantify the uncertainty of predictions, so that human curators can decide whether to manually re-inspect an automatically flagged GOA. We explore this by analyzing the change in a model’s *F*_1_ considering various uncertainty values as the threshold for positive prediction. Uncertainty quantification was introduced in Section 3.3. The calculation of *F*_1_ under a certain uncertainty threshold is described as follows: Assume the uncertainty threshold τ=1.0, the true label of an instance is OS. Then, the prediction of this instance is treated as TP if the predicted label is OS and τ<1.0. The prediction of this instance is treated as FN if the predicted label is not OS. The prediction of an OB instance is treated as false OS instance (FP) if the predicted label is OS and τ<1.0. We calculate eight groups of TP, FN, FP for each type of inconsistency and each instance under different values of *τ* {0.5, 0.75, 1, 1.25, 1.5, 1.75, 2, 2.3}. Each group of TP, FN, FP will be used to calculate *F*_1_.

## 4 Results

Table 4 shows that PubMedBERT and GNN-BERT, fine-tuned with ID samples, are reasonable in distinguishing four types of inconsistencies. They all significantly outperform the Baseline in detecting over-specific inconsistencies. Generally, the detection of IG inconsistency is poor ($F_{1}<0.3$). The rule Baseline outperforms two machine-learning-based approaches in distinguishing over-broad instances. However, it is not promising for the detection of over-specific instances and is not applicable to detect ‘irrelevant GO mention’ and ‘incorrect gene association’ instances, as discussed in Section 3.2.

**Table 4.** Performance of baseline, PubMedBERT and GNN-BERT in discriminating inconsistencies including over-specific (OS), over-broad (OB), irrelevant GO mention (IM) and incorrect gene (IG). The bold values indicate the highest performance in detection of each type of inconsistency.

	Baseline	PubMedBERT		GNN-BERT	
		ID	OOD	ID	OOD
OS					
* Precision*	0.18	0.30	0.41	**0.54**	0.45
* Recall*	1.00	0.52	0.42	**0.62**	0.56
* F*_1_	0.30	0.38	0.41	**0.58**	0.50
OB					
* Precision*	**0.75**	0.53	0.49	0.52	0.53
* Recall*	0.56	**0.73**	0.69	0.69	0.65
* F*_1_	**0.64**	0.61	0.57	0.59	0.58
IM					
* Precision*	NA	0.38	0.33	**0.42**	0.33
* Recall*	NA	0.73	0.71	**0.83**	0.79
* F*_1_	NA	0.50	0.45	**0.56**	0.47
IG					
* Precision*	NA	0.18	0.15	**0.24**	0.17
* Recall*	NA	0.19	0.27	**0.40**	0.35
* F*_1_	NA	0.18	0.20	**0.30**	0.23

ID, the model is fine-tuned using in-distribution samples and OOD indicates the model is fine-tuned with out-of-distribution samples; NA, not applicable (the Baseline method does not recognize gene mentions and has zero *Recall* as it considers every GO mention as negative regardless of true IM inconsistency).

GNN-BERT outperforms PubMedBERT in detecting over-specific instances (+ 0.2 in *F*_1_) and achieves comparable performance (–0.02) in detecting over-broad instances. In particular, GNN-BERT outperforms PubMedBERT in discriminating the semantics of GO specificity, as supported by the more competitive performance on detecting over-specific instances. This type of instance requires detection of smaller semantic differences than over-broad instances as mentioned in Section 3.1.

**Fig. 4.**
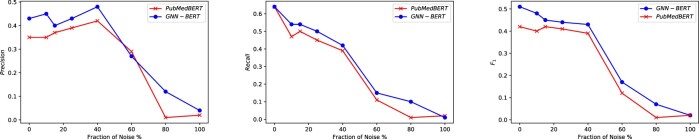
Change of performance of PubMedBERT and GNN-BERT on the test set with respect to the fraction of noisy samples in the training set

Figure 4 shows that both PubMedBERT and GNN-BERT are robust to noisy samples, with good maintenance of *Precision*, *Recall* and *F*_1_ on the test set when the level of noise is below 40% during fine-tuning stage. Besides, GNN-BERT has slightly outperformed PubMedBERT in terms of robustness to noisy samples, potentially indicating its feasibility in combining GO specificity knowledge during consistency inference.

Table 4 demonstrates that both PubMedBERT and GNN-BERT are robust to OOD samples in detecting over-broad instances but are challenged in the detection of other GOA inconsistencies. PubMedBERT gained slight improvement in *F*_1_ when detecting over-specific (+ 0.03) and ‘incorrect gene’ (+ 0.02) instances.

**Fig. 5.**
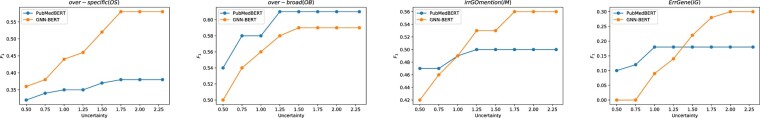
Change of *F*_1_ of PubMedBERT and GNN-BERT in discriminating four types of GOA inconsistency with respect to prediction uncertainty

Figure 5 illustrates the correlations between model’s uncertainty and *F*_1_ on the detection of different typed inconsistencies. The performance of both PubMedBERT and GNN-BERT can be maintained at a promising level when the tolerance towards prediction uncertainty is set at a high value (*τ >* 1.75). GNN-BERT outperforms PubMedBERT on the detection of over-specific instances regardless of the prediction uncertainty. PubMedBERT outperforms GNN-BERT on the detection of IM and IG instances when only low uncertainty predictions are treated as positives. PubMedBERT is more competitive than GNN-BERT in detecting over-broad inconsistencies. The detection of over-broad inconsistency outperforms over-specific inconsistency for both PubMedBERT and GNN-BERT. We assume it is due to over-broad instances were synthesized with greater semantic distance, as introduced in Section 3.1, allowing both models more capability to distinguish between inconsistency and self-consistency.

## 5 Discussion

In this article, we have demonstrated that automatic detection of inconsistencies in GOA is feasible. Our presentation of three automatic approaches for effective detection of four realistic types of inconsistencies shows good levels of effectiveness, providing a practical basis for flagging suspicious records to human curators for manual review if any inconsistency is detected among GO term, gene product and literature.

### 5.1 General observations

Relative to prior research on this topic (Chen *et al.*, 2021), we have extended the formal specification of GOA inconsistency modeling from the consistency of a pair—a GO term and the corresponding literature evidence for the GO annotation—into a triplet, with the addition of gene product to the input. This means that the consistency of gene function knowledge is checked in the context of a specific gene product, GO term and literature evidence. To explore modeling of this extended task, we have constructed a dataset from biological database records, which includes not only the inconsistencies reflected in both datasets, including over-specific (OS) and irrelevant GO mention (IM) but also two types of GOA inconsistency not previously considered, over-broad (OB) and incorrect gene (IG). Based on an informal survey of database curators, we understand that these two new types of inconsistencies often occur in databases but have not been covered in prior work. We also excluded two inconsistencies considered in that prior work, unsupportive evidence and erroneous evidence code, as they were confirmed by curators from 14th Annual biocuration conference (https://www.biocuration.org/14th-annual-biocuration-conference-virtual/) to be rare.

Our results indicate strong feasibility for the real-world application of the automatic methods we introduce. The proposed GNN-BERT model combines GO specificity knowledge as part of the GOA inconsistency inference. In the systematic comparison with PubMedBERT and Baseline methods shown in Table 4, we can observe the strong competitiveness of the GNN-BERT model in discriminating GO terms with smaller semantic distance, and its strong robustness to noise.

The analysis in Figure 4 indicates that PubMedBERT and GNN-BERT are both robust to moderate noise (<40%) during fine-tuning. However, the existence of noisy samples in the training set eventually reduces model performance on the inference of GOA inconsistency. The trend indicates that the performance of PubMedBERT and GNN-BERT could be directly improved by providing a small collection of expert-reviewed instances during fine-tuning, without the requirement of altering the architecture of the models. Accordingly, the fluctuations of performance seen in Figure 4 (cf. a linear degradation in performance with additional noise) may support the conclusion that these types of simulated inconsistencies do exist in current databases, as a small injection of noise may have inadvertently flipped a true inconsistency to a consistency in the training set, or vice versa.

The two machine-learning approaches outperform the rule-based Baseline in detecting over-specific instances even they were fine-tuned with OOD samples. The OOD samples have a more negative impact to GNN-BERT than PubMedBERT overall. This suggests that both proposed models would reach higher reliability if they were able to be fine-tuned with a small amount of manually verified GOA records for the target species during application in a specific curation workflow.

Both learned models are feasible in real-world application settings in the face of prediction uncertainty while there is variability in their relative performance ranking for specific GOA inconsistencies under different uncertainty thresholds.

The results of the Baseline on over-broad cases demonstrate the value of incorporating existing automatic approaches to identify GO terms (e.g. ConceptMapper) into the two-step rule-based GOA inconsistency detection model. This strategy may be extended to convert a broad range of existing biological concept or entity recognition models to inconsistency detection, such as using PubTator (Wei *et al.*, 2013) to identify genes directly mentioned in the literature evidence and applying a check for (in)consistency with the target gene product.

### 5.2 Error analysis

Previous work by Chen *et al.* (2021) proposed a linguistic test suite to understand the relationship between certain superficial linguistic features of GO terms and prediction errors in inconsistency detection for relating GOA to evidence text. However, this approach is not generally meaningful to biological database curators as it does not explain errors from a more biological perspective. To address this issue, here we provide qualitative case studies with the objective of identifying biologically interpretable causes of prediction errors. These cases demonstrate clear directions for future improvement and can be directly leveraged as use cases for real-world application settings.

**Case 1 [GO depth]**. We find both PubMedBERT and GNN-BERT are ineffective for classifying inconsistent instances where the associated GO terms are either very shallow or very deep (i.e. roots or leaves, or terms directly connected to roots or to leaves) on the GO DAG. This is probably caused by annotation bias in the training set, where many GOA utilize broad GO terms without sufficient representation of specificity in gene functional information (Camon *et al.*, 2005; Škunca *et al.*, 2012). Thus, the model may label a rarely occurring GO term as over-specific and a frequently occurring GO term as over-broad. Apart from this, we find both models failed to discriminate inconsistencies between over-specific and IM, or over-broad and IM. However, we consider this type of error to be tolerable when the models are applied in real-world application settings. This is because all suspicious records flagged by the automatic models as inconsistent will be eventually forwarded to human curators for further review regardless of their inconsistency types. And furthermore, these cases are not likely to cause false consistent predictions (FNs) for the proposed models. Thus, it will not impact the models’ reliability in discriminating inconsistent GOA from consistent records.

**Case 2 [Related gene]**. We find both PubMedBERT and GNN-BERT cannot discriminate inconsistencies for related genes. We find the outcome of the two models remain unchanged when the gene in the input is replaced with another related gene, such as ‘elt-2|GeneID: 181250’ is replaced with ‘elt-1|GeneID: 177794’ in a GOA that associates with ‘PMID: 17183709’ and ‘GO: 0050829’. We assume this type of error is probably caused by the tokenization process of two models. The pre-trained BERT tokenizer will split gene variant symbols into text pieces during preprocessing. Because most of the gene symbols are out-of-vocabulary to PubMedBERT and GNN-BERT. For example, ‘elt-2’ will be tokenized into ‘el, ##t, -, 2’, which cannot represent the full semantics of gene variants. Thus, both models failed to discriminate inconsistencies regarding the gene variant. A possible solution is to apply ensemble models to PubMedBERT or GNN-BERT specifically for recognizing gene variants.

**Case 3 [Gene synonyms]**. We find neither model can infer gene synonyms between the evidence and gene symbol. For example, both models incorrectly predict the following consistent instance as an inconsistency: ‘Tinman/Nkx2-5 acts via miR-1 and upstream of Cdc42 to regulate heart function across species (PMID: 21690310)’, ‘FSD1 (GeneID: 79187)’, ‘heart process (GO: 0003015)’, where gene ‘FSD1’ is also known as ‘miR-1’ but was not explicitly mentioned within the evidence. A potential solution is to apply gene normalization to the input instance before feeding into PubMedBERT or GNN-BERT, to resolve synonyms and other name variations.

**Case 4 [Titles]**. We find both models can be slightly improved in *F*_1_ when they are fed with only abstract text (no title) as evidence in the input. On the contrary, we find both models are negatively impacted in *F*_1_ when they are only provided with title as evidence in the input. However, some older primary literature does not have abstract content published (e.g. PMID: 4186849). The reliability of the predictions is below expectation on these GOA records.

**Case 5 [Related GO terms]**. We find both models under-perform in discriminating the nuance between semantic relatedness and consistency of GO terms. For example, the gene functions ‘cytokinasis (GO: 0000910)’ and ‘chromosome segregation (GO: 0007059)’ are semantically related to each other but not consistent to the same GOA instance. However, we find both models cannot distinguish inconsistency when ‘cytokinasis’ is replaced with ‘chromosome segregation’, such as in the following instance: ‘The CeCDC-14 phosphatase is required for cytokinesis in the *C.elegans* (PMID: 12213836)’, ‘cdc-14 (GeneID: 173945)’.

## 6 Conclusion

Automatic GOA consistency assurance is important to maintain currency and quality of the information of gene function knowledge in modern organism databases and may improve the efficiency of manual GOA curation. We explored the feasibility of automatic methods assisting the validation of literature-based GOA records. We formally identified four major types of inconsistent GO annotations that reflect the major GO annotation quality concerns for the GO curation community. We proposed a novel and efficient approach based on automatic methods and comparatively explored two models derived from benchmark natural language processing technologies to automatically detect self-consistent and these four major types of inconsistent GO annotations, evaluated using a new automatically generated data set that fully simulates realistic GOA records. While there is substantial room for improvement in the models, particularly in detecting the irrelevant GO mention and incorrect gene inconsistencies, the results are sufficiently promising to warrant further work on automatic GOA inconsistency detection.

In future, our methodological work will focus on improving the detection of GOA inconsistencies relating to incorrect gene products (IG). We will experiment with ensemble models for improving the performance of our models in detecting GOA instances of special cases. We will extend the detection of inconsistency of gene function knowledge from a single GOA record to GO causal activity model (Thomas *et al.*, 2019).

To better understand the value of our approach in the context of quality control of GO annotations in biological databases, we also plan to apply an end-to-end GOA inconsistency detection model on an existing biological database and to explore the implications of the approach for biological database curation processes.

## Data Availability

**The data and code underlying this article are available in Github at**: https://github.com/jiyuc/AutoGOAConsistency

## Funding

This work was supported by an Australian Research Council Discovery Project grant, [DP190101350 to K.V., N.G. and J.Z.]. The funding body played no role in the design or execution of the research.

*Conflict of Interest*: none declared.
